# Nondestructive quantification of internal raster path for additively manufactured components via ultrasonic testing

**DOI:** 10.1038/s41598-024-61416-5

**Published:** 2024-05-19

**Authors:** Atik Amin, David A. Jack, Pruthul Kokkada Ravindranath, Trevor J. Fleck

**Affiliations:** Department of Mechanical Engineering, Baylor University, 1301 S University Parks Dr, Waco, TX 76798 USA

**Keywords:** Additive manufacturing, Fused filament fabrication, Raster orientation, Ultrasonic nondestructive testing, C-scan, 3D printing, Mechanical engineering, Characterization and analytical techniques, Mechanical properties

## Abstract

This work investigates the viability of discerning the raster pattern of additively manufactured components using high frequency ultrasonic nondestructive testing. Test coupons were fabricated from poly cyclohexylenedimethylene terephthalate glycol using the fused filament fabrication process, in which layers were deposited at various predetermined raster angles. Each printed part was scanned using spherically focused, high-resolution, ultrasonic transducers of various peak frequencies between 7.5 and 15 MHz. From the captured waveform data, images are extracted to observe the raster pattern in a layer-by-layer manner, with the results from the 10 MHz element yielding the best performance. An in-house MATLAB script was developed to analyze the transducer signal to investigate C-scan images at various depths throughout the component. From the resulting C-scan images, one can consistently identify the proper raster orientation within 2°–4° in each of the first 10 deposited layers, with the accuracy decreasing as a function of depth into the component. Due to signal attenuation, there is insufficient data at depths beyond the 11th and 12th layer, to properly analyze the present data sets accurately. Validation was performed using X-ray computed tomography scans to demonstrate the accuracy of the ultrasonic inspection method.

## Introduction

Additive Manufacturing (AM) is an increasingly popular set of processes for fabricating complex three dimensional objects and applications of this practice are providing advanced product design in the manufacturing industry. In fused filament fabrication (FFF), the additive manufacturing technology employed in the present study, a fusion-based material extrusion process is carried out by melting a polymer filament in a heated nozzle and depositing the molten layers onto previously deposited layers or directly onto the heated built plate^1^. Continued advances with CAD, CAM and CNC cutting technologies have helped to grow the rapid prototyping process and make it more efficient while allowing for tighter manufacturing tolerances (see e.g.,^2,3^). The molten layers cool rapidly and harden, forming a three-dimensional part established by a pre-defined nozzle path (see e.g.,^1,3,4^). Many materials used for FFF are commercially available plastics, with two of the most commonly used being acrylonitrile butadiene styrene (ABS) and polylactic acid (PLA)^5^. There are other polymers available in filament form such as, polycarbonate (PC), polycaprolactone (PCL), polyetherimide (PEI), polyether ether ketone (PEEK) and polyether ketone ketone (PEKK). When fabricating a component using the FFF process, the nozzle deposits individual lines or beads on the print bed. These individual beads are known as rasters, with raster orientation, also called build orientation, being defined as the print bead direction with respect to a given axis. This orientation may change both within a deposited layer and between layers based upon the design needs. The final part performance of the additively manufactured part will be a function of the choice of material systems and the printing orientation, among other process parameters.

As such, researchers have demonstrated that the raster orientation of the component changes the fabricated part strength (see e.g.,^6,7^). Several process parameters often considered in design are the build orientation, layer thickness, raster width, raster angle, air gap, etc., affecting both the final part dimensionality and distortion within the part (see e.g.,^8^). The deleterious effects of these parameters often result in an AM component that has poorly defined anisotropic characteristics^9^. One of the most influential of these parameters, the aforementioned raster orientation, plays a significant role as it affects the build time, the required support structure and dimensional accuracy (see e.g.,^10^). In addition, literature has shown that varying the raster orientation has a significant impact on the resultant mechanical, fracture and failure properties of a 3D printed object (see e.g.,^11^). For example, in^12^ a part with a 0°/90° repeating raster angle, the tensile and bending strengths are, respectively 56.6 MPa and 56.1 MPa, whereas the same part and material system with a 45°/ − 45° repeating raster angle able yielded a tensile strength and bending strength of, respectively, 43.3 MPa and 43.2 MPa. It was observed the raster oriented parallel to the load direction created the samples with the highest hardness and stiffness (see e.g.,^12–15^). Yap et al.^16^ investigated the effects of raster angles and orientations on the elastic properties of a 3D printed PC-ABS material. They presented a method to determine the effective orthotropic elastic constants by NDT testing and found to be in good agreement with the experimental results for the simple orientations studied (all 0° and 0°/90° repeating).

Prajapati et al.^17^ identified diminished properties in the build direction will occur for various orientations. In Prajapeti et al. they demonstrated a technique to mitigate the loss in performance through a thermal annealing process. Es-Said et al.^18^ also studied the interlayer bonding and demonstrated that because of the weak interlayer adhesion for their test samples fabricated with a 0° orientation, rupture occurred at the individual layer steps. The work of Es-Said et al. also discussed the phase change occurring when the semi-molten filament extrudes and solidifies in a chamber during deposition and variations in the material contraction or the respective layers increase the likelihood of triggering a fragile interlayer connection. As such, the significant mechanical properties, such as tensile strength and flexural strength, are functions of the relative orientation patterns. There is a considerable breadth of literature in the scientific community focused on the structural impact of the raster orientation within the additively manufactured component highlighting the importance of non-destructively identifying the as manufactured raster orientation and relative alignments of individual rasters (see e.g.,^19^).

To date, there has been limited work investigating the uses of ultrasonic inspection of FFF components, most of which has focused on either manufacturing or in-service damage. Among all NDT techniques it was found computed tomography (CT) and ultrasonic testing (UT) are the most effective inspection methods (see e.g.,^20^). Lee et al.^21^ presented an ultrasonic approach to identify various embedded voids within an additively manufactured component with a specific focus on porosity. Na and Oneida^22^ presented a through-transmission immersion approach for inspection of FFF components and studied various defects, such as delamination and embedded voids using a reference standard approach. Poudel et al.^23^ used a pulse-echo UT testing method coupled with digital image correlation to characterize the defects within carbon fiber reinforced epoxy (CFRP) panels during loading. Phased array ultrasound (PAUT) was employed to identify service induced damage within a CFRP^24^. Fayazbakhsh et al.^25^ used a high frequency PAUT method to identify defects, specifically inter-raster voids, within a part fabricated using the FFF technique. In addition, they used structural testing to investigate the relationship between the gap width and tensile properties, using the results from the ultrasonic testing to differentiate defective regions within the specimen and correlated the results to the structural testing results. Machado et al.^26^ incorporated two different UT testing methods, air-coupled ultrasound and active transient thermography, and were able to characterize the defects within a curved part and compared the results to their numerical simulation. Jin et al.^27^ used an ultrasonic imaging technique to characterize the density within a 3D printed part manufactured with acrylonitrile butadiene styrene (ABS) and found the accumulated lateral and axial capabilities of the imaging method to be effective for in situ inspection. Camineroa et al.^24^ investigated barely visible impact damage using ultrasound and identified a complex network of matrix cracking and delamination inside the composite parts. High resolution UT inspected data was used in conjunction with the Fast Fourier Transform to characterize the probabilistic strength, stiffness and failure analysis of carbon fiber composite laminates (see e.g.,^28,29^), and the method introduced in^29^ is extended in the present study to investigate a neat polymer system fabricated using FFF. Jones^30^ described the strength of a composite structure is dependent on the orientation of its each lamina. A method of transformation between the stress and strain is shown in^30^ as different material orientations will have different principal material coordinates and the overall strength of the material will vary as a function of the orientation. The influence of carbon fiber orientations on the mechanical properties are discussed in^31,32^ where both unidirectional and mixed-isotropic configurations were used.

The work herein presents a method using high-resolution ultrasound (UT) to identify the as-manufactured raster orientation. This is an extension of the authors’ preliminary study in^33^ that provided an overview of the inspection results for a variety of material systems without validation. The earlier work in^33^ utilized test coupons printed with High Temperature Nylon (HTN) and Carbon Fiber filled Polyethylene Terephthalate (PET-CF). In the work the results from HTN the 18th raster layer could be observed, whereas the PET-CF coupons only the 10th raster layer could be identified. Given the increased usage of AM components, being able to identify and reverse engineer the effective as manufactured behavior is becoming increasingly important. In addition to quantifying the raster orientation of FFF components, this study gives insights into which inspection parameters are well suited for qualifying FFF components. First, standard coupons were manufactured on an industrial-scale FFF printer with a variety of raster orientations. These components were then inspected using high-resolution UT. Using the UT data, an in-house MATLAB script was developed to analyze the transducer signal to investigate C-scan images—throughout the component. These measurements were then compared with the as-designed orientation and validated against X-ray computed tomography (CT) images.

## Research and experimental methodology

### Material selection and printing

A polymer filament with a diameter of 1.75 mm was selected for this study and printed using an industrial-scale FFF system (Essentium HSE 180-HT) with a 0.8 mm nozzle diameter. All parts were 76.2 mm × 76.2 mm (3 inches × 3 inches) in the planar dimension, with a part height of 6.35 mm (0.25 inches). Slicing of the CAD model was performed using Simplify 3D and process parameters were adjusted to that of the manufacture’s recommended specifications, as shown in Table 1, with a nozzle temperature of 340 °C and a print speed of 100 mm/s. All parts were fabricated using Poly Cyclohexylenedimethylene Terephthalate Glycol (PCTG) (Essentium, 1.75 mm, black). A total of nine samples are included in this study. Of the nine samples, they are subdivided into three sample types, A, B and C. The orientation of each sample type is provided in Table 1. Each set sample type, A, B and C, has a unique pattern of raster orientations. Sample type A has alternating raster layers at 90° offset pairs. This is done to prevent the overlap of individual layers and to mitigate warping. Sample type B uses a quasi-isotropic layup with layers offset by 60° intervals. This also has the same strategy as A as to balance the sub-layers to mitigate warping effects. Conversely, sample type C uses a more random set of raster orientations. The samples are labeled as A_0X, B_0X and C_0X, where the “X” is 1, 2 or 3 referring to the first, second or third sample of the given type. For example, sample B_02 would be the second sample of orientation sequence B. Prior to printing the part, the filament was dried, per the manufacturer’s recommended procedures, for 4 h at a temperature of 65–70 °C^34^. Before using the FFF system, the standard manufacturer process recommendations, such as automatically leveling the print bed and checking first layer uniformity, were used.

**Table 1:** Sample type summary.

Sample type	Print orientation (°)	Layer height (mm)	Number of layers	Infill (%)
A	[0,90,10,100,20,110,30,120,40, 130,50,140,60,150,70,160,80,170]	0.35	18	100
B	[0,60,120,20,80,140,40,100,160, 60,120,180,80,140,200,100,160,220]	0.35	18	100
C	[0,90,45,135,20,110,70,160,50, 140,85,175,25,115,10,100,110,180]	0.35	18	100

### Ultrasonic inspection experimental setup

**Figure 1:**
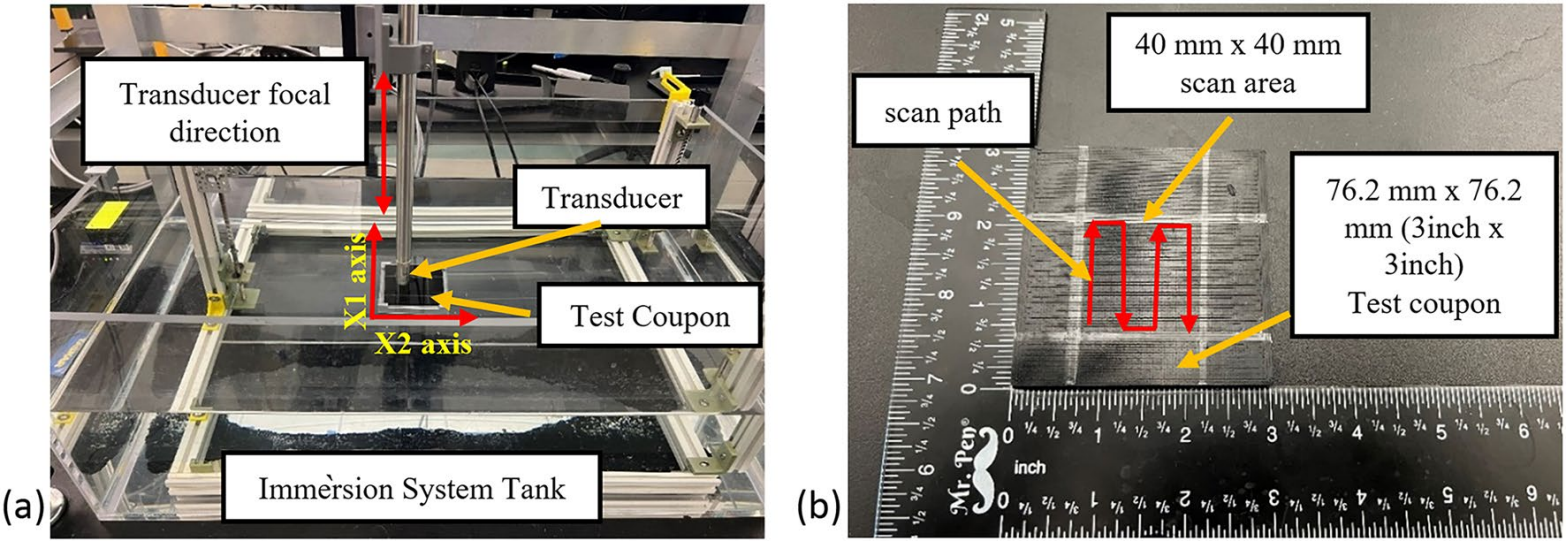
Experimental setup for ultrasound investigation, (**a**) custom immersion system and (**b**) schematic of scan area.

For nondestructive inspection (NDI), the custom immersion ultrasonic system shown in Fig. 1a is used. The immersion system uses Velmex translation stages with 1/800th mm spatial resolution and a Focus PX pulser/receiver operating in full-waveform data capture mode. Spherically focused transducers operating in pulse-echo mode with a 38.1 mm (1.5 inch) nominal focal length are used in this study with frequencies of 7.5 MHz, 10 MHz and 15 MHz. A digitizer operating at 100 MHz was used to capture the waveform and a pulse voltage of 190 V was used for all three studies. A pulse width of 1/*f* is used where *f* is the transducer frequency. The choice of 7.5–15 MHz transducers was made based upon a balance between layer thickness and signal attenuation. Generally, transducers with higher peak frequencies are capable of capturing finer features or defects in the component being inspected. Unfortunately, as the transducer peak frequency increases, the rate of attenuation increases, causing poor signal-to-noise ratios deeper into the part. The reason for surveying multiple transducers is to check what level of frequency can transmit through the layers properly and provide viable C-scan images. The immersion ultrasonic system used contains an acrylic tank filled with water to fully immerse the sample being inspected. The samples are placed on the fixture shown in Fig. 1 to align the sample in the (*x*_1_, *x*_2_) coordinate system. The transducer, shown in Fig. 1a, is attached to a search tube that can be moved up and down to focus on different planes within the part being inspected, with all scans in the present study focusing on the midplane of the component. The resolution for the *x*_1_ and *x*_2_ directions were set to 0.1 mm and all movement is controlled by a custom in-house software interface created within the MATLAB environment. The raster scan path is depicted in Fig. 1b within the 40 mm × 40 mm scan area region to indicate how the data was collected.

### X-ray CT experimental setup

X-ray Computed Tomography (CT) is used to validate the UT measurements. A North Star Imaging Inc. (NSI) X-3000 industrial X-Ray CT inspection system is used, which transmits a conical X-ray beam. This conical beam is generated from an electron beam of 75 kV voltage with a 750 µA current. A voxel (3D pixel) size of 41.5 µm was achieved using a 3 × magnification scale. A total of 1440 images were extracted from the 3D scan with 10 frames per image on average. Reconstruction and analysis of the individual x-ray scans is performed using the NSI efX-CT reconstruction software (version 2.2.5.2).

## Analysis

**Figure 2:**
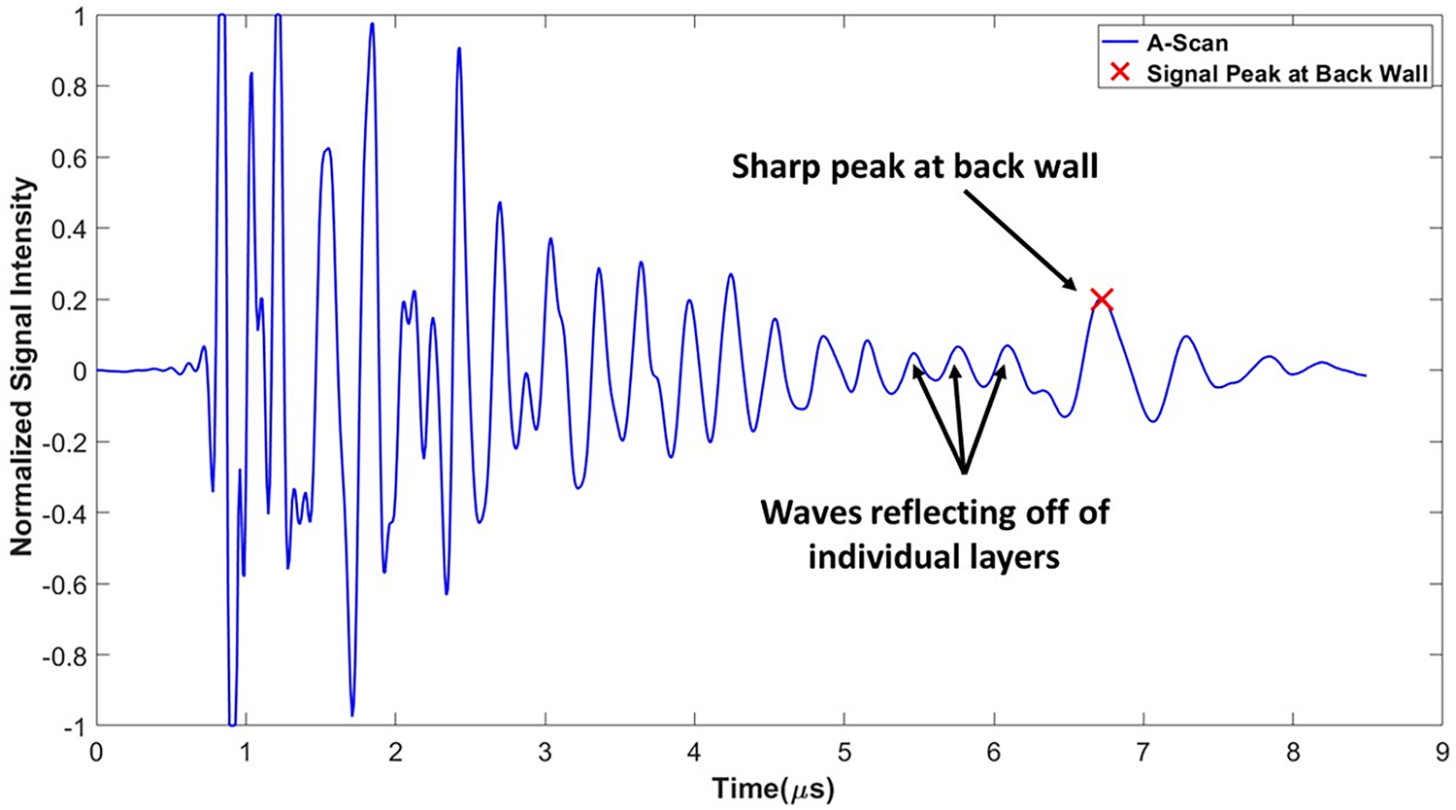
A-scan image for ${\tilde{\mathcal{F}}}\left( {x_{1} ,x_{2} ,\tilde{t}} \right)$ at (*x*_1_, *x*_2_) = (200 mm, 200 mm). from Part A_01 using a 10 MHz transducer.

**Figure 3:**
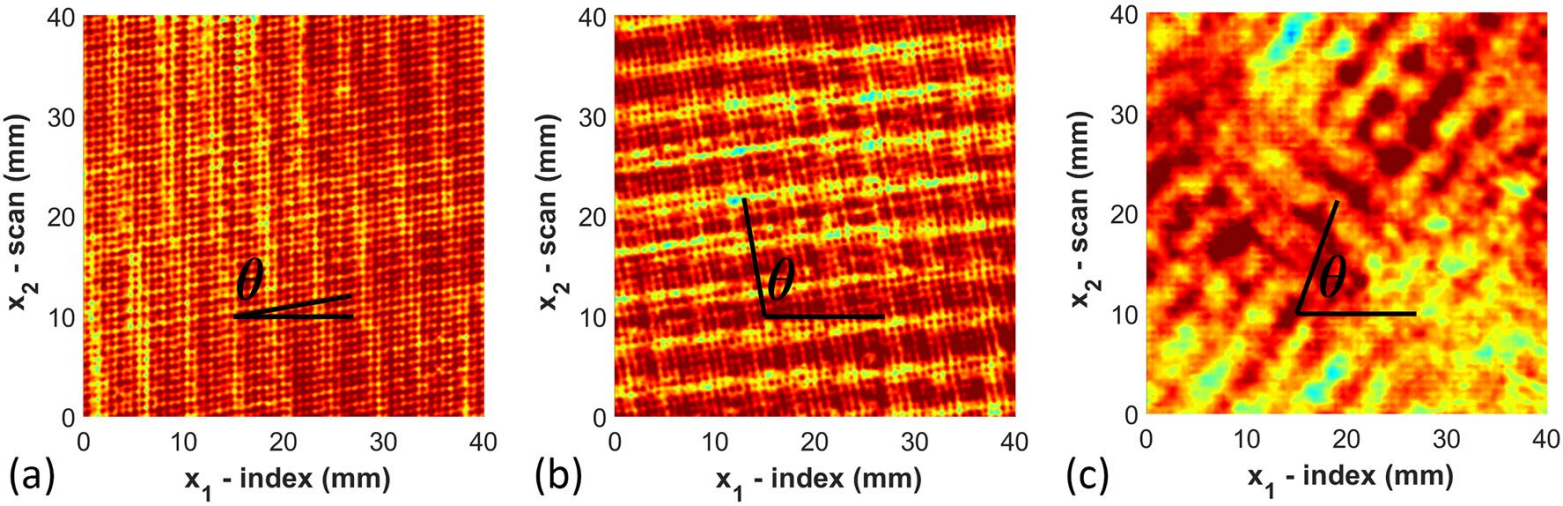
Raster orientation of individual layer for PCTG Part A_01 using a 10 MHz transducer extracted from the ultrasonic C-scan data for the (**a**) 3rd layer, (**b**) 4th layer, and (**c**) 15th layer.

Once the full waveform is captured from the ultrasound inspection data, the results are saved in an array ${\mathcal{F}}\left( {x_{1,k} ,x_{2,l} ,t} \right)$. This array contains the amplitude of the captured acoustic waveform as a function of time *t* and spatial position (*x*_1_, *x*_2_) at discrete points $\left( {x_{1,k} ,x_{2,l} } \right)$. The data is then shifted in time $\tilde{t}\left( {x_{1} ,x_{2} } \right) = t - t_{0} \left( {x_{1} ,x_{2} } \right)$, where $t_{0} \left( {x_{1} ,x_{2} } \right)$ is the moment in time when the front wall echo is received by the firing transducer. The function ${\mathcal{F}}\left( {x_{1,k} ,x_{2,l} ,\tilde{t}\left( {x_{1,k} ,x_{2,l} } \right)} \right)$ is then smoothed using a spatial Gaussian filter as^35^ as

1 $${\tilde{\mathcal{F}}}\left( {x_{1,k} ,x_{2,l} ,\tilde{t}} \right) = \frac{1}{{2\pi \sigma_{{x_{1} }} \sigma_{{x_{2} }} }}\mathop \smallint \limits_{ - \infty }^{\infty } \mathop \smallint \limits_{ - \infty }^{\infty } e^{{\frac{{ - \left( {\tilde{x}_{1} - x_{1,k} } \right)^{2} }}{{2\sigma_{{x_{1} }}^{2} }}}} e^{{ - \frac{{\left( {\tilde{x}_{2} - x_{2,l} } \right)^{2} }}{{2\sigma_{{x_{2} }}^{2} }}}} {\mathcal{F}}\left( {\tilde{x}_{1} ,\tilde{x}_{2} ,\tilde{t}\left( {\tilde{x}_{1} ,\tilde{x}_{2} } \right)} \right) d\tilde{x}_{1} d\tilde{x}_{2}$$

where ${\tilde{\mathcal{F}}}\left( {x_{1,k} ,x_{2,l} ,\tilde{t}} \right)$ uses the nearest neighbors to smooth the full waveform based upon the spread of the distribution parameters $\sigma_{{{\text{x}}_{1} }}$ and $\sigma_{{{\text{x}}_{2} }}$. In the present study $\sigma_{{{\text{x}}_{1} }}$ and $\sigma_{{{\text{x}}_{2} }}$ were set to 0.3 mm (three times the step size of 0.1 mm). A typical waveform after spatial smoothing is shown in Fig. 2, is saved for analysis. The waveform in Fig. 2, a single A-scan at (*x*_1_, *x*_2_) = (200 mm, 200 mm), shows the front wall occurring near 1 μs and the back wall near 7 μs. Each of the individual peaks are closely correlated to individual raster layers, with each layer causing both a reflection back to the transmitting transducer and a refraction deeper into that part. From the single A-scan image it is not possible to identify anything about the directionality of the individual raster layers. The individual A-scans are then gathered, and a single C-scan corresponding to a specific depth is plotted as shown in Fig. 3. These C-scans are surface plots of ${\tilde{\mathcal{F}}}\left( {x_{1,k} ,x_{2,l} ,\tilde{t}} \right)$ for a fixed value of $\tilde{t}$. The value for $\tilde{t}$ is selected to correlate to specific depths within the part, thus based upon the value for $\tilde{t}$ and the known speed of sound of the material, one can extract the raster orientation of a given layer from the associated C-scan. Figure 3 contains the C-scan from three different depths within part A_01 corresponding to the 3rd, 4th, and 15th deposited layer. From the images, one can observe the raster orientation of the given layer. For example, for the 4th deposited layer, the orientation measured is 100° and the designed orientation is also 100°. The orientation is identified by selecting two points from the C-scan of any of the identified rasters and calculating the slope. This process is completed for three properly identified rasters within the 40 mm × 40 mm scan area as shown in Fig. 1 for every layer extracted from the C-scan. The rasters were randomly selected in three different regions of the scan region; and the average measurement of orientations of the three rasters is reported as *θ*_*NDT*_ listed in Table A1 shown in appendix. This process is then repeated at each depth within the C-scan waveform stack, and the measured orientations from each of the raster layers are tabulated. The layers are identified using Fig. 2 where the signal peaks denote the sound wave as it passes through each layer and weakens as it transmits through the depth of the part. The sharp peak at the end implies that the signal has reached the back wall. The speed of sound was measured manually using the measured distance through which the signal passes within the part divided by the signal duration. The average speed of sound for the as manufactured samples of PCTG was measured as 2333 m/s.

## Results and discussion

### Ultrasonic inspection

After scanning the parts in the immersion tank, a manual procedure of finding the raster orientation of each layer was performed where two points were selected along the length of the same identified bead using the *getpts* function in MATLAB after importing the images of individual layers with the function *imshow*. The rasters were chosen from the top corner, midpoint, and bottom corner within each layer. The positions of the two selected points from the selected rasters were used to compute the slope and subsequently the orientation of the raster within the layer. This process was repeated three times at the locations discussed and the average of the three measurements is the value reported as *θ*_*NDT*_. The method was repeated for all nine parts used in the experiment and the standard deviations were measured.

**Figure 4:**
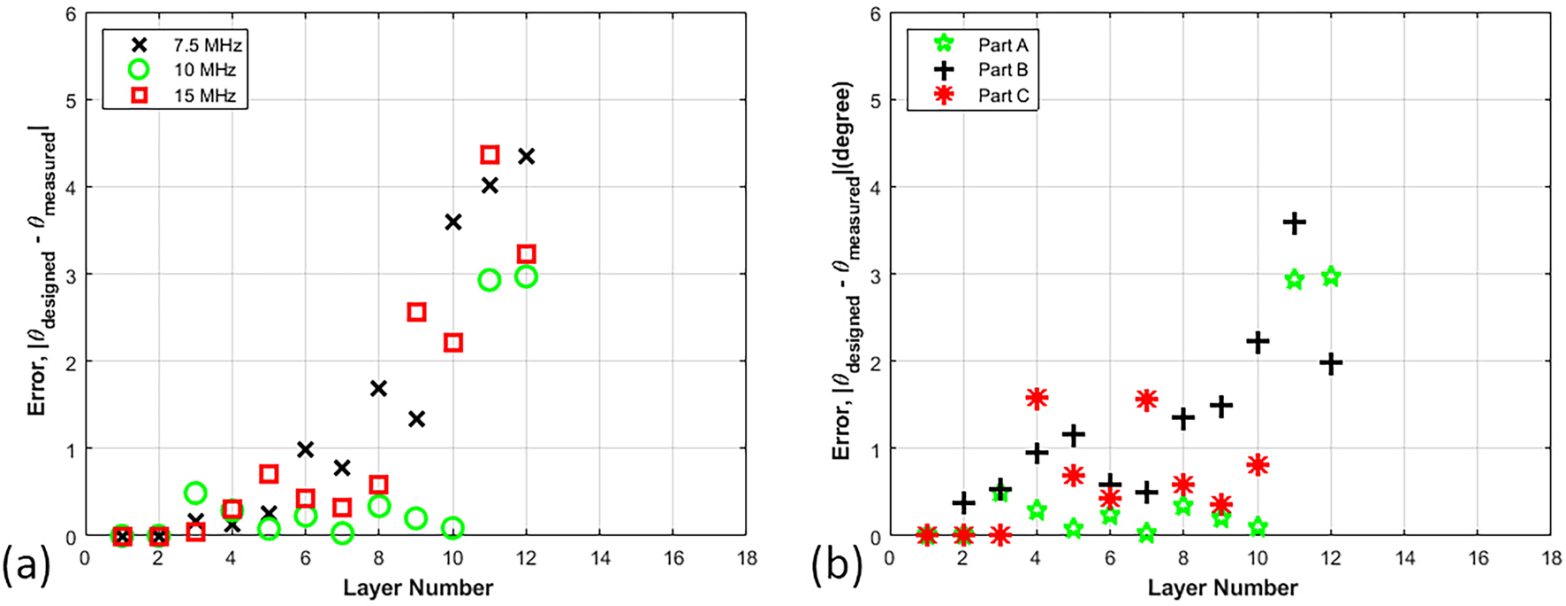
Errors measured against the number of layers (**a**) Part A scanned with 7.5, 10 and 15 MHz transducers (**b**) Parts A, B and C scanned with 10 MHz transducer.

Figure 3 shows the sliced C-scan images from selected layers. From these images, it is observed that the layers closest to the transducer result in a well-defined raster orientation. However, as the signal passes through the thickness of the sample, attenuation occurs and individually imaged rasters become less visible. Layers 3 and 4 contain the raster orientation 10° & 100° respectively as shown in Fig. 3a,b by visual inspection, and it is possible to visually identify the orientation of the rasters. Conversely, for Fig. 3c, which corresponds to the 15th layer, there is no clear discernable orientation to the rasters but it is blurry from a visual perspective. The results obtained after the ultrasonic analysis of all samples (A_01, A_02, A_03) of part A are shown in Table A1. The table includes the designed orientation along with the measured orientation obtained using 7.5 MHz, 10 MHz and 15 MHz transducers along with the absolute value of the error (Fig. 4). The error is defined as

2 $$E_{rr} = \left| {\theta_{original} - \theta_{NDT} } \right|$$

where *θ*_*NDT*_ is the angle measured from the individual C-scans. Conversely, a higher frequency will result in higher resolution through the thickness of a part. Transducers higher than 15 MHz were considered but were unable to identify the back-wall of the 6.35 mm thick part, whereas using frequencies lower than 7.5 MHz the current authors were unable to differentiate the individual layers within the part. The complete set of results are numerically provided in Table A1 given in the appendix and plotted in Fig. 4a. In Fig. 4a the error is plotted as a function of layer depth for each of the three transducer frequencies. Notice that each of the transducer frequencies was effective at identifying the orientation up to the 12th layer, with an error less than 4°. Looking more closely at the data, the 10 MHz transducer has an error less than 2° for the first 10 layers, and this trend continues across all parts investigated. For part A_01 the orientation can be estimated for several layers after the 10th layer from the 10 MHz transducer and the signal starts to attenuate significantly after the 13th to 14th layer. Past the 14th layer it is unclear what subtle features from the sectioned C-scan correspond to the current raster layer as opposed to what is an internal echo from an earlier raster layer.

The results for the 10 MHz transducer across all three parts are provided in Fig. 4b where the error plot against the number of layers for three different parts are shown. Signals from the 10 MHz transducer while scanning all samples of part A show the best performance in terms of accuracy and penetration depth, as noted for Fig. 4a, and this trend continued for all samples from parts B and C. In all cases, the error in determining the orientation for the first 9 layers is less than 1.5° and there is difficulty in identifying the layers past the 12th lamina. Table A2 in appendix shows the numerical results from all three part types scanned with the 10 MHz transducer. It should be noted that, for part C after the 10th layer, it was difficult to identify any orientation from the captured waveform, hence the error is not presented in Table A2 past the 10th layer. Observe from Fig. 4b that for each of the parts the results from the 10 MHz transducer has an error that increases with increasing depth, but it remains less than 3.6°.

**Figure 5:**
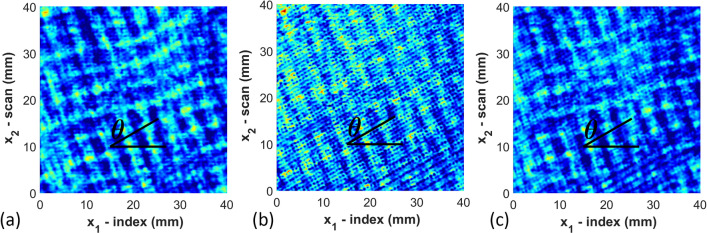
Comparison of C-scans of layer 07 of Part A_01 using (**a**) 7.5 MHz (**b**) 10 MHz (**c**) 15 MHz transducers.

The images in Fig. 5 present three different images of Layer 07 of Part A_01 at each of the investigated frequencies. The rasters are orientated at 30° for the 7th layer. Figure 5a indicates the test coupon when scanned with the 7.5 MHz transducer, and the orientations are very difficult to identify from the image, whereas for the results from the 10 MHz transducer, shown in Fig. 5b, the individual rasters are identifiable. Similarly, the raster can be observed in the data from the 15 MHz transducer in Fig. 5c, but it is less clear than that of the signal generated by the 10 MHz transducer. Based upon the results from part A and additional internal studies that included parts B and C, the waveforms generated and captured by the 10 MHz transducer yields the highest accuracy. This conclusion is specific to the material system studied and the specific raster height, and should not be directly extrapolated to other parts. Based upon in-house related studies for other materials on our printer, the 10 MHz seemed to work reasonably well across the material systems we studied and may serve as a reasonable starting point for future investigations.

In this study different transducers were used to investigate how well the signal waves of varying frequency can detect the internal raster path of a 3D printed part. Apart from using transducers with varying frequency levels, it is necessary to determine the layer height and thickness of the 3D printed test coupon. When the speed of sound or material is unknown, the A-scan can be used to locate the backwall via the required time the signal to transmit through the part, which can be cross checked against the time value required for passing through each layer. Scan resolution is another important parameter that can be considered. For this study the scan resolution is taken as 0.1 mm. The smaller the scan resolution is taken the higher the precision of the result we will get from the ultrasonic inspection.

**Figure 6:**
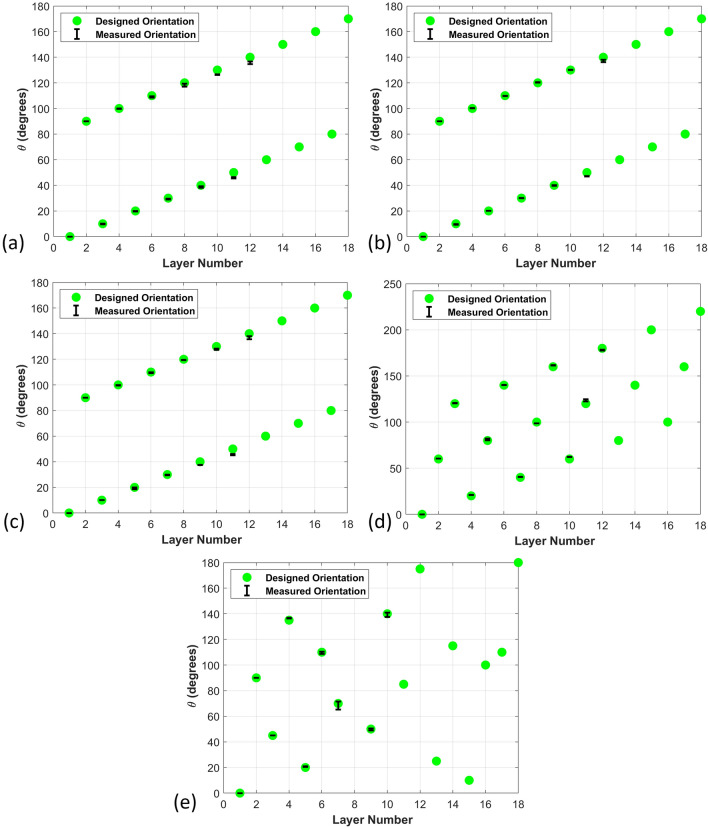
Graphical representation of designed and measured orientation against layer number (**a**) Part A—7.5 MHz transducer (**b**) Part A—10 MHz transducer (**c**) Part A—15 MHz transducer (**d**) Part B—10 MHz transducer (**e**) Part C—10 MHz transducer.

Figure 6 represents the accuracy of the manual orientation measurements compared to the designed orientations. In many cases the error is less than a degree up to the 7th or 8th layer, and up to the 10th layer this error is typically less than 4 degrees with no clear difference between the various parts with the different layer orientations. The value for the error bars in the figures come from the standard deviation over the three samples taken for each part.

### CT validation

**Figure 7:**
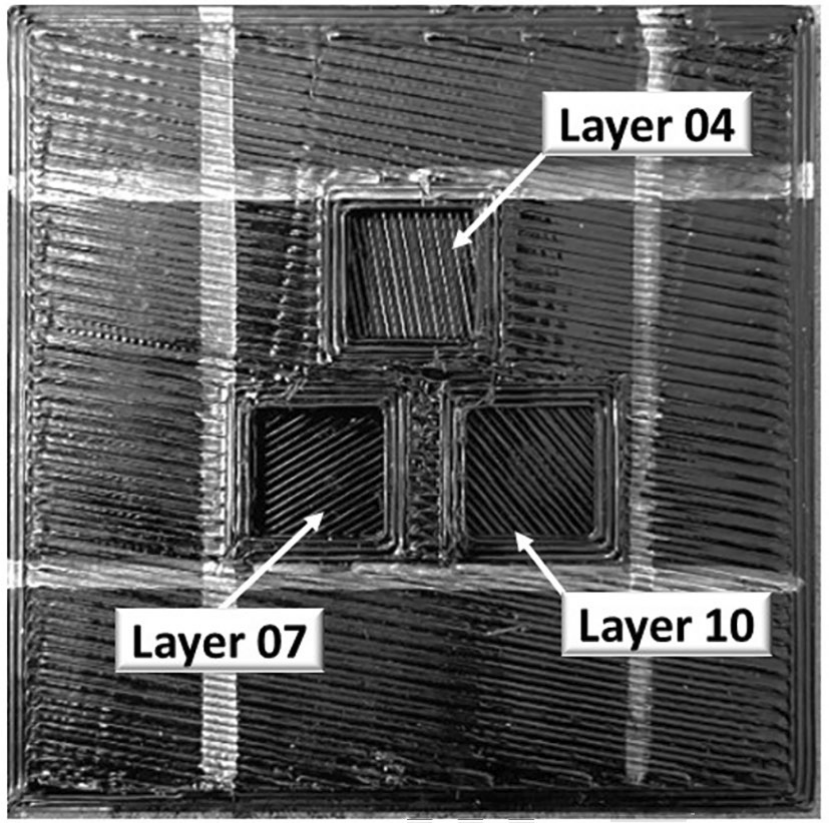
Modified test coupon for the validation of raster orientation at layer 04, 07 and 10.

**Figure 8:**
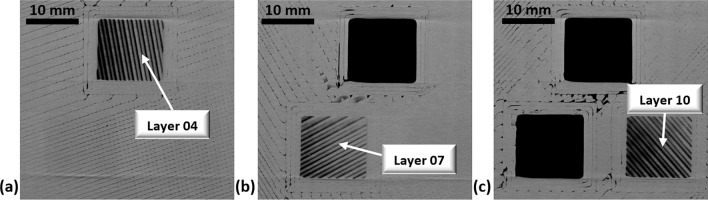
Extracted images of Layer 04, 07 and 10 at their individual depths using X-ray CT.

To validate the results provided in Section "Ultrasonic inspection", a part was reprinted with the PCTG material following the orientation pattern A and CT testing is performed on the part to extract the individual raster orientations. To validate the CT methodology itself, a modification to the design for part A is performed to expose three different internal rasters. To do this, three different square cut extrusions were made within the 40 mm × 40 mm scan area at three different layer heights. The goal was to check the raster orientation visually and against the CT observations. Figure 7 shows the orientations of Layer 04, 07 and 10 printed according to the given direction in the slicing software representing 100°, 30° and 130°. This part was then scanned using X-ray CT, and individual layers were extracted from the reconstructred CT data set. The X-ray CT layer images shown in Fig. 8 extracted from the NSI efX-CT reconstruction software was found to match the optically observed orientations of the printed part shown in Fig. 7. Figure A1, given in the appendix shows the summary of the measurements of raster orientation from X-ray CT. Specifically, Figure A1(b) shows the errors for each layer are within 1° of the as designed raster orientation.

**Figure 9:**
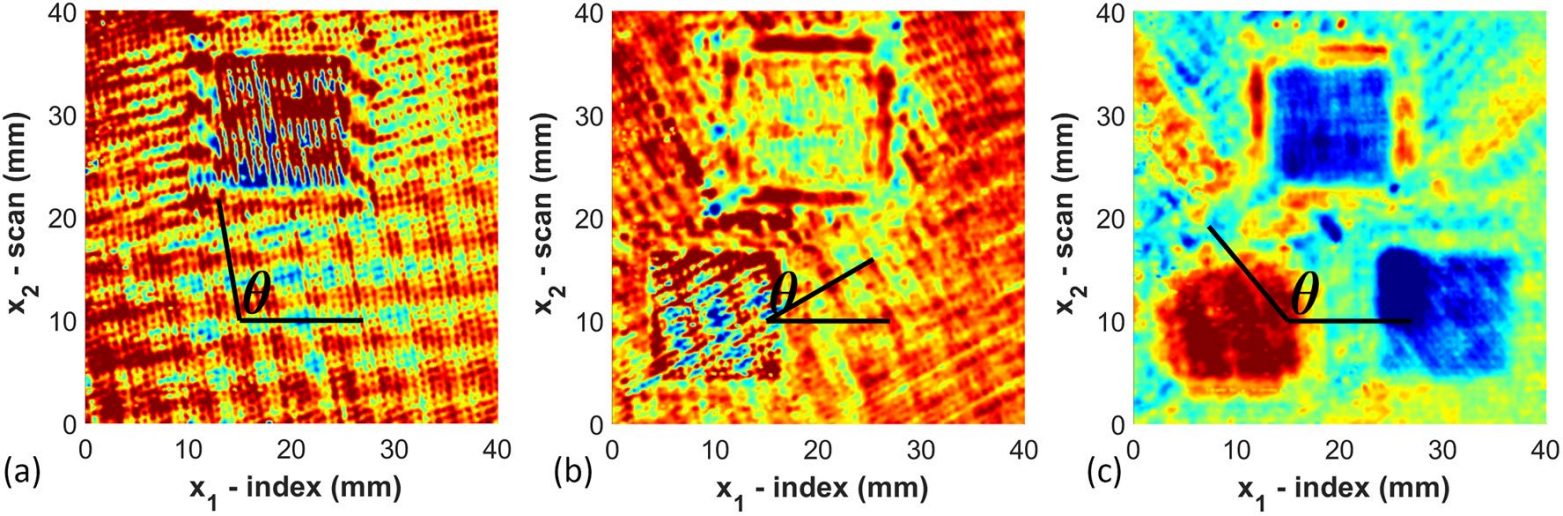
Extracted images of layer 04, 07 and 10 at their individual depths using ultrasonic waveform results.

After completing the CT scan, the sample was placed inside the immersion tank and ultrasonic testing was performed. Figure 9a shows layer 04, and it demonstrates that the inspected orientation using UT matches the as designed orientation and the CT identified orientation from Fig. 8a. As the ultrasound signal passes through the individual layers, its intensity is weakened. Therefore, by layer 07 it is becomes difficult to properly quantify the appropriate angle within the square section (Fig. 9b). By the 10th raster layer, shown in Fig. 9c, the orientation is nearly impossible to identify visually. However, there is still enough signal resolution and contrast that the orientation of 130° can be observed within the square region, representing the 10th layer.

In the present study the separation between A-scans was 0.1 mm over the 40 mm × 40 mm scan region of the test coupon. The time required for each scan was 33–35 min. In contrast the effective pixel pitch for the CT system was 0.042 mm and the scan region of interest was the entire 76.2 mm × 76.2 mm object. The CT imaging took over 2 h and the subsequent reconstruction another 2 h.

## Conclusion

Fused filament fabrication is becoming a widely used technique in the aerospace industry. At present, most of the parts are manufactured for rapid prototyping, however, efforts are being made to implement FFF components in functional applications. As a result, detecting the quality of internal features and/or the geometry of FFF components is a growing need. The work described in the research paper uses ultrasonic testing to inspect and quantify internal features of FFF coupons. This study showed that the raster orientation can be measured with an accuracy of 2°–4° up to the 12th layers into a component, with the inspection performed using the 10 MHz spherically focused transducer being the most effective. These measurements were validated using X-ray CT. As a result, a cost-effective method of investigating the raster orientation using the presented UT method is feasible, and future studies could focus on automated methods for quantification of the raster orientation.

### Supplementary Information

Supplementary Information.

## Data Availability

The datasets used and/or analyzed during the current study available from the corresponding author on reasonable request.

## References

[1] Jiménez M, Romero L, Domínguez IA, Espinosa MDM, Domínguez M (2019). Additive manufacturing technologies: An overview about 3D printing methods and future prospects. Complexity.

[2] Wong KV, Hernandez A (2012). A review of additive manufacturing. ISRN Mech. Eng..

[3] Parupelli SK, Desai S (2019). A comprehensive review of additive manufacturing (3D printing): Processes, applications and future potential. Am. J. Appl. Sci..

[4] Coogan TJ, Kazmer DO (2020). Prediction of interlayer strength in material extrusion additive manufacturing. Addit. Manuf..

[5] Kantaros A, Karalekas D (2013). Fiber Bragg grating based investigation of residual strains in ABS parts fabricated by fused deposition modeling process. Mater. Des..

[6] Jiang D, Smith DE (2017). Anisotropic mechanical properties of oriented carbon fiber filled polymer composites produced with fused filament fabrication. Addit. Manuf..

[7] Vega V, Clements J, Lam T, Abad A, Fritz B, Ula N, Es-Said OS (2011). The effect of layer orientation on the mechanical properties and microstructure of a polymer. J. Mater. Eng. Perform..

[8] Ahn SH, Montero M, Odell D, Roundy S, Wright PK (2002). Anisotropic material properties of fused deposition modeling ABS. Rapid. Prototyp. J..

[9] Sood AK, Ohdar RK, Mahapatra SS (2010). Parametric appraisal of mechanical property of fused deposition modelling processed parts. Mater. Des..

[10] Pandey PM, Venkata Reddy N, Dhande SG (2007). Part deposition orientation studies in layered manufacturing. J. Mater. Process. Technol..

[11] Lorenzo-Bañuelos M, Díaz A, Cuesta II (2020). Influence of raster orientation on the determination of fracture properties of polypropylene thin components produced by additive manufacturing. Theor. Appl. Fract. Mech..

[12] Wu W, Geng P, Li G, Zhao D, Zhang H, Zhao J (2015). Influence of layer thickness and raster angle on the mechanical properties of 3D-printed PEEK and a comparative mechanical study between PEEK and ABS. Materials.

[13] Durgun I, Ertan R (2014). Experimental investigation of FDM process for improvement of mechanical properties and production cost. Rapid. Prototyp. J..

[14] Sood AK, Ohdar RK, Mahapatra SS (2012). Experimental investigation and empirical modelling of FDM process for compressive strength improvement. J. Adv. Res..

[15] Tymrak BM, Kreiger M, Pearce JM (2014). Mechanical properties of components fabricated with open-source 3-D printers under realistic environmental conditions. Mater. Des..

[16] Yap YL, Toh W, Koneru R, Lin K, Yeoh KM, Lim CM, Lee JS, Plemping NA, Lin R, Ng TY, Chan KI, Guang H, Chan WYB, Teong SS, Zheng G (2019). A non-destructive experimental-cum-numerical methodology for the characterization of 3D-printed materials—polycarbonate–acrylonitrile butadiene styrene (PC-ABS). Mech. Mater..

[17] Prajapati H, Chalise D, Ravoori D, Taylor RM, Jain A (2019). Improvement in build-direction thermal conductivity in extrusion-based polymer additive manufacturing through thermal annealing. Addit. Manuf..

[18] Es-Said OS, Foyos J, Noorani R, Mendelson M, Marloth R, Pregger BA (2000). Effect of layer orientation on mechanical properties of rapid prototyped samples. Mater. Manuf. Process..

[19] Bharvirkar, M., Nguyen, P., & Pistor, C. *Thermo-mechanical Analysis of Parts Fabricated via Fused Deposition Modeling*. 10.26153/tsw/824

[20] Honarvar F, Varvani-Farahani A (2020). A review of ultrasonic testing applications in additive manufacturing: Defect evaluation, material characterization, and process control. Ultrasonics.

[21] Lee, J., Hasanian, M., Saboonchi, H., Baechle, M., & H. Taheri. Ultrasonic evaluation of polymer additively manufactured parts for defect inspection and structural integrity assessment. in *SPIE-Intl Soc Optical Eng* 70 (2020). 10.1117/12.2572463.

[22] Na JK, Oneida EK (2018). Nondestructive evaluation method for standardization of fused filament fabrication based additive manufacturing. Addit. Manuf..

[23] Poudel, A., Li, S., & Chu, T. P. *An Intelligent Systems Approach for Detecting Delamination Defects Due to Impact Damage in CFRP Panel by Using Ultrasonic Testing Speckle Pattern Development and Applications of Digital Image Correlation (DIC) on Adhesive Joints View Project NDE of Additive Manufacturing View Project* (2011). https://www.researchgate.net/publication/277010881.

[24] Caminero MA, García-Moreno I, Rodríguez GP, Chacón JM (2019). Internal damage evaluation of composite structures using phased array ultrasonic technique: Impact damage assessment in CFRP and 3D printed reinforced composites. Compos. B Eng..

[25] Fayazbakhsh K, Honarvar F, Amini H, Varvani-Farahani A (2021). High frequency phased array ultrasonic testing of thermoplastic tensile specimens manufactured by fused filament fabrication with embedded defects. Addit. Manuf..

[26] Machado, M. A. et al. *Inspection of Composite Parts Produced by Additive Manufacturing: Air-Coupled Ultrasound and Thermography*. https://www.researchgate.net/publication/335662161

[27] Jin Y, Walker E, Heo H, Krokhin A, Choi TY, Neogi A (2020). Nondestructive ultrasonic evaluation of fused deposition modeling based additively manufactured 3D-printed structures. Smart Mater. Struct..

[28] Blackman, N. PhD dissertation, *Evaluation of Carbon Fiber Laminates via the Use of Pulse-Echo Ultrasound to Quantify Ply-Stack Orientation and Manufacturing Defects* (2021). https://hdl.handle.net/2104/11715

[29] Rahul, K., & Jack, D. A. Investigating the impact from ultrasonic testing uncertainty in quantifying ply stack orientation on the probabilistic failure envelope.in *SPE ACCE Conference* 2022 September 7–9. https://speautomotive.com/wp-content/uploads/2022/10/Impact-from-UT-Uncertainty-in-Quantifying-Ply-Stack-Orientation-on-Probablistic-Failure-Envelope_RAHUL.pdf

[30] Jones, R. M. *Mechanics of Composite Materials* 2nd ed (CRC Press, 1999). 10.1201/9781498711067

[31] Parmiggiani, A., Prato, M., & Pizzorni, M. Effect of the Fiber Orientation on the Tensile and Flexural Behavior of Continuous Carbon Fiber Composites Made via Fused Filament Fabrication (n.d.). 10.1007/s00170-021-06997-5/Published.

[32] Pathak AK, Garg H, Subhedar KM, Dhakate SR (2021). Significance of carbon fiber orientation on thermomechanical properties of carbon fiber reinforced epoxy composite. Fibers Polym..

[33] Amin, A., Jack, D. A., & Flect, T. J. Quantitative inspection of internal raster orientation of additively manufactured components via ultrasonic nondestructive testing. in *SPE ACCE Conference 2022 September 7–9*. https://speautomotive.com/wp-content/uploads/2022/10/Quantitative-Inspection-of-Internal-Raster-Orientation-of-Additively-Manufactured-Components-via-Ultrasonic-Nondestructive-Testing.pdf

[34] Essentium PCTG Technical Data Sheet. https://www.essentium.com/wp-content/uploads/2020/08/TDS-Essentium-PCTG-Z_v1.0-Minus-3D.pdf.

[35] Blackman NJ, Jack DA, Blandford BM (2021). Improvement in the quantification of foreign object defects in carbon fiber laminates using immersion pulse-echo ultrasound. Materials.

