# Supplementary material for: Nondestructive quantification of internal raster path for additively manufactured components via ultrasonic testing
Source: Sci Rep. 2024 May 19;14:11416. doi: 10.1038/s41598-024-61416-5 (PMC11102913; doi:10.1038/s41598-024-61416-5)
Supplement: Supplementary file 1 — Supplementary Information. [file 41598_2024_61416_MOESM1_ESM.docx]

**Appendix**

Table A 1 - Errors measured for all samples of Part A scanned with 7.5, 10 and 15 MHz transducers with all units in degrees.

| **Raster Layer** | $\theta_{original}$ | **7.5 MHz** | | **10 MHz** | | **15 MHz** | | |
| --- | --- | --- | --- | --- | --- | --- | --- | --- |
|  |  | $\theta_{NDT}$ | $Err_{7.5}$ | $\theta_{NDT}$ | $Err_{10}$ | $\theta_{NDT}$ | $Err_{15}$ |  |
| 1 | 0 | 0 | 0 | 0 | 0 | 0 | 0 |  |
| 2 | 90 | 90 | 0 | 90 | 0 | 90 | 0 |  |
| 3 | 10 | 9.84 | 0.16 | 9.51 | 0.49 | 10.05 | 0.05 |  |
| 4 | 100 | 99.87 | 0.13 | 100.29 | 0.29 | 99.69 | 0.31 |  |
| 5 | 20 | 19.74 | 0.26 | 20.08 | 0.08 | 19.29 | 0.71 |  |
| 6 | 110 | 109.02 | 0.98 | 109.77 | 0.23 | 109.58 | 0.42 |  |
| 7 | 30 | 29.22 | 0.78 | 30.03 | 0.03 | 29.67 | 0.33 |  |
| 8 | 120 | 118.31 | 1.69 | 120.34 | 0.34 | 119.41 | 0.59 |  |
| 9 | 40 | 38.65 | 1.34 | 39.80 | 0.20 | 37.43 | 2.57 |  |
| 10 | 130 | 126.40 | 3.60 | 130.10 | 0.10 | 127.79 | 2.21 |  |
| 11 | 50 | 45.98 | 4.02 | 47.073 | 2.93 | 45.63 | 4.37 |  |
| 12 | 140 | 135.66 | 4.34 | 137.03 | 2.97 | 136.77 | 3.23 |  |
| 13 | 60 | --- | --- | --- | --- | --- | --- |  |
| 14 | 150 | --- | --- | --- | --- | --- | --- |  |
| 15 | 70 | --- | --- | --- | --- | --- | --- |  |
| 16 | 160 | --- | --- | --- | --- | --- | --- |  |
| 17 | 80 | --- | --- | --- | --- | --- | --- |  |
| 18 | 170 | --- | --- | --- | --- | --- | --- |  |

Table A 2 – Errors measured for different parts scanned with 10 MHz transducer

| **Raster Layer** | **Part A** | | **Part B** | | **Part C** | | |
| --- | --- | --- | --- | --- | --- | --- | --- |
|  | $\theta_{original}$ | $Err$ | $\theta_{original}$ | $Err$ | $\theta_{original}$ | $Err$ |  |
| 1 | 0 | 0 | 0.0 | 0 | 0.0 | 0 |  |
| 2 | 90 | 0 | 60.0 | 0.38 | 90.0 | 0 |  |
| 3 | 10 | 0.49 | 120.0 | 0.53 | 45.0 | 0 |  |
| 4 | 100 | 0.29 | 20.0 | 0.96 | 135.0 | 1.58 |  |
| 5 | 20 | 0.08 | 80.0 | 1.16 | 20.0 | 0.69 |  |
| 6 | 110 | 0.23 | 140.0 | 0.59 | 110.0 | 0.42 |  |
| 7 | 30 | 0.03 | 40.0 | 0.49 | 70.0 | 1.57 |  |
| 8 | 120 | 0.34 | 100.0 | 1.35 | 160.0 | 0.59 |  |
| 9 | 40 | 0.20 | 160.0 | 1.50 | 50.0 | 0.36 |  |
| 10 | 130 | 0.10 | 60.0 | 2.23 | 140.0 | 0.82 |  |
| 11 | 50 | 2.93 | 120.0 | 3.59 | 85.0 | --- |  |
| 12 | 140 | 2.97 | 180.0 | 1.99 | 175.0 | --- |  |
| 13 | 60 | --- | 80.0 | --- | 25.0 | --- |  |
| 14 | 150 | --- | 140.0 | --- | 115.0 | --- |  |
| 15 | 70 | --- | 200.0 | --- | 10.0 | --- |  |
| 16 | 160 | --- | 100.0 | --- | 100.0 | --- |  |
| 17 | 80 | --- | 160.0 | --- | 110.0 | --- |  |
| 18 | 170 | --- | 220.0 | --- | 180.0 | --- |  |
|  |  |  |  |  |  |  |  |

Figure A 1 – X-ray CT raster orientation quantification, (a) As designed and measured orientation, (b) Error between the as designed orientation and the X-ray CT characterized orientation.


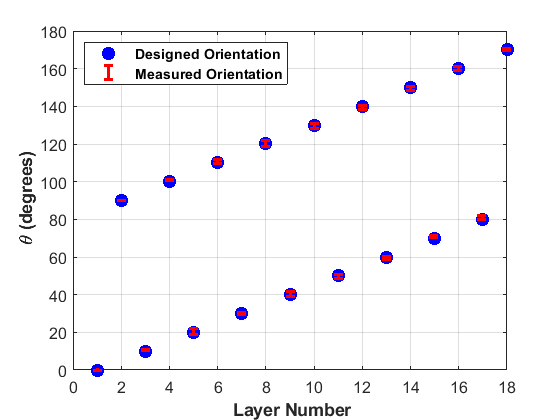


**(a)**


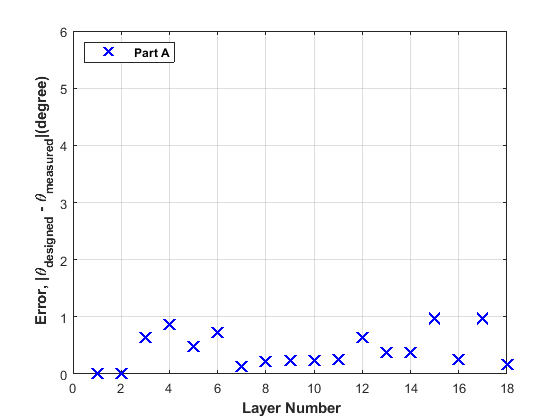


**(b)**
